# Delivery of small interfering RNAs in human cervical cancer cells by polyethylenimine-functionalized carbon nanotubes

**DOI:** 10.1186/1556-276X-8-267

**Published:** 2013-06-06

**Authors:** Yuan-Pin Huang, I-Jou Lin, Chih-Chen Chen, Yi-Chiang Hsu, Chi-Chang Chang, Mon-Juan Lee

**Affiliations:** 1 Department of Cosmetics and Fashion Styling, Cheng Shiu University, Kaohsiung 83347, Taiwan; 2 Department of Bioscience Technology, Chang Jung Christian University, No. 1 Changda Rd., Gueiren District, Tainan City 71101, Taiwan; 3 Department of Obstetrics and Gynecology, E-Da Hospital, Kaohsiung 82445, Taiwan; 4 Graduate Institute of Medical Sciences, Chang Jung Christian University, Tainan 71101, Taiwan

**Keywords:** SWNTs, MWNTs, PEI, Small interfering RNA, Gene delivery

## Abstract

Carbon nanotubes are capable of penetrating the cell membrane and are widely considered as potential carriers for gene or drug delivery. Because the C-C and C=C bonds in carbon nanotubes are nonpolar, functionalization is required for carbon nanotubes to interact with genes or drugs as well as to improve their biocompatibility. In this study, polyethylenimine (PEI)-functionalized single-wall (PEI-NH-SWNTs) and multiwall carbon nanotubes (PEI-NH-MWNTs) were produced by direct amination method. PEI functionalization increased the positive charge on the surface of SWNTs and MWNTs, allowing carbon nanotubes to interact electrostatically with the negatively charged small interfering RNAs (siRNAs) and to serve as nonviral gene delivery reagents. PEI-NH-MWNTs and PEI-NH-SWNTs had a better solubility in water than pristine carbon nanotubes, and further removal of large aggregates by centrifugation produced a stable suspension of reduced particle size and improved homogeneity and dispersity. The amount of grafted PEI estimated by thermogravimetric analysis was 5.08% (*w*/*w*) and 5.28% (*w*/*w*) for PEI-NH-SWNTs and PEI-NH-MWNTs, respectively. For the assessment of cytotoxicity, various concentrations of PEI-NH-SWNTs and PEI-NH-MWNTs were incubated with human cervical cancer cells, HeLa-S3, for 48 h. PEI-NH-SWNTs and PEI-NH-MWNTs induced cell deaths in a dose-dependent manner but were less cytotoxic compared to pure PEI. As determined by electrophoretic mobility shift assay, siRNAs directed against glyceraldehyde-3-phosphate dehydrogenase (siGAPDH) were completely associated with PEI-NH-SWNTs or PEI-NH-MWNTs at a PEI-NH-SWNT/siGAPDH or PEI-NH-MWNT/siGAPDH mass ratio of 80:1 or 160:1, respectively. Furthermore, PEI-NH-SWNTs and PEI-NH-MWNTs successfully delivered siGAPDH into HeLa-S3 cells at PEI-NH-SWNT/siGAPDH and PEI-NH-MWNT/siGAPDH mass ratios of 1:1 to 20:1, resulting in suppression of the mRNA level of GAPDH to an extent similar to that of DharmaFECT, a common transfection reagent for siRNAs. Our results indicate that the PEI-NH-SWNTs and PEI-NH-MWNTs produced in this study are capable of delivering siRNAs into HeLa-S3 cells to suppress gene expression and may therefore be considered as novel nonviral gene delivery reagents.

## Background

Carbon nanotubes (CNTs) are cylindrical structures formed by graphite sheets with a diameter in the nanometer range and tens to hundreds of micrometers in length [1]. They can be categorized into single-wall carbon nanotubes (SWNTs) and multiwall carbon nanotubes (MWNTs), according to the number of concentric layers of graphite sheets.

Carbon nanotubes are being extensively studied as carriers for gene or drug delivery [2-5]. In order to provide functional groups for the binding of plasmid DNAs, small interfering RNAs (siRNAs), or chemical compounds and to reduce the potential toxicity of pristine carbon nanotubes, functionalization of carbon nanotubes is necessary for their biomedical applications [6-10]. After complexed with nucleotides or chemicals through either covalent or noncovalent binding, functionalized carbon nanotubes may then enter cells by endocytosis [3,11,12] or by penetrating directly through the cell membrane [13-15].

To serve as carriers for nonviral gene delivery, as opposed to viral transfection which applies viral vectors to achieve high transfection efficiency, carbon nanotubes are often functionalized with cationic molecules or polymers in order to interact electrostatically with negatively charged siRNAs or plasmid DNAs [7,9,16-19]. SWNTs and MWNTs chemically modified with amino groups were capable of delivering plasmid DNAs into A549, HeLa, and CHO cell lines [18,19]. MWNTs functionalized with polycationic dendron may enhance siRNA delivery and gene silencing *in vitro*[9]. Furthermore, positively charged SWNTs in complex with telomerase reverse transcriptase siRNAs were shown to suppress tumor growth in animal studies [17]. Intratumoral administration of cytotoxic siRNAs delivered by amino-functionalized MWNTs successfully suppressed tumor volume in animal models of human lung cancer [20].

Polyethylenimine (PEI), a cationic polymer synthesized in linear or branched form with various molecular weights, is used in several studies to provide a high density of cations on the surface of carbon nanotubes [21,22]. It was shown that PEI-grafted MWNTs improve the expression of plasmid DNA in human embryonic kidney (HEK 293) and human lung epithelial (A549) cells [22,23]. Shortened MWNTs of 200 nm in length covalently modified with branched PEI of low molecular weight (600 Da) deliver siRNAs with higher efficacy than a lipid vehicle [21]. Successful delivery of siRNA to human prostate cancer PC-3 cells by PEI-functionalized SWNTs was also reported [24]. Moreover, PEI-modified SWNTs were shown to provide the substrate for neurite outgrowth and branching [25].

Despite extensive applications, PEI, itself a reagent for nonviral transfection, is cytotoxic, and chemical modification of PEI is required to improve its application as a transfection reagent [23,26,27]. It is therefore expected that functionalization of carbon nanotubes with PEI would not only increase their biocompatibility but also reduce the toxicity of PEI. Nevertheless, contradictory conclusions on the toxicity and transfection efficiency of PEI-functionalized carbon nanotubes compared to pure PEI were presented in the literature [21,23,24,28]. In this study, SWNTs and MWNTs were functionalized with PEI for the delivery of siRNAs. The properties and efficiencies of PEI-functionalized SWNTs and MWNTs as nonviral transfection reagents were compared, and whether the functionalization procedure reduces the cytotoxicity of PEI was discussed.

## Methods

### Materials

SWNTs of 2 to 10 nm in diameter were purchased from Sigma-Aldrich, St. Louis, MO, USA. MWNTs of 20 to 40 nm in diameter were produced by Seedchem Company, Melbourne, Australia. Branched PEI with an average *M*_W_ of approximately 25,000 and an average *M*_n_ of approximately 10,000 was manufactured by Sigma-Aldrich.

### PEI functionalization of carbon nanotubes

Carbon nanotubes were covalently modified with PEI by following the direct amination procedure in the literature [29,30]. SWNTs or MWNTs (500 mg) were mixed with 2.5 g PEI in 50 ml dimethylformamide. The mixture was sonicated for 30 min and stirred at 50°C for 3 days, followed by filtration through a 0.2-μm nylon membrane (Millipore Co., Billerica, MA, USA). The resulting PEI-functionalized carbon nanotubes (PEI-NH-CNTs) were washed successively with 1 M HCl, 1 M NaOH, double-distilled water (ddH_2_O), and methanol, and dried under vacuum. PEI-NH-CNTs were then resuspended in ddH_2_O at a concentration of 1 mg/ml, sonicated for 15 min, and centrifuged at 3,000 rpm for 30 min. The supernatant was stored at 4°C and used in the following studies.

### Characterization of PEI-NH-CNTs

The difference in morphology between pristine and PEI-functionalized carbon nanotubes was examined by transmission electron microscopy (TEM; 2000FX, JEOL Ltd., Akishima, Tokyo, Japan) and scanning electron microscopy (SEM; JSM-6500F). Fourier transform infrared (FTIR) spectra of pristine and PEI-functionalized carbon nanotubes were obtained with a PerkinElmer (Branford, CT, USA) Spectrum 100 FTIR spectrometer. The amount of grafted PEI in PEI-NH-CNTs was determined by thermogravimetric analysis (TGA) using a PerkinElmer Pyris 1 TGA instrument under nitrogen atmosphere over a temperature range from 50°C to 800°C at a heating rate of 10°C/min. The particle size and zeta potential of PEI-NH-CNTs were determined by dynamic light scattering using Zetasizer Nano ZS system (Malvern Instruments, Worcestershire, UK).

### Electrophoretic mobility shift assay

Dharmacon siGENOME GAPD control siRNA (glyceraldehyde 3-phosphate dehydrogenase siRNA (siGAPDH)) was purchased from Thermo Fisher Scientific, Waltham, MA, USA. The PEI-NH-CNT/siGAPDH complex was formed by incubating 0 to 80 μg of PEI-NH-CNTs with 0.5 μg siGAPDH at various mass ratios (0:1 to 160:1) in serum-free RPMI-1640 medium on ice for 1 h. The complex was then mixed with SYBR Green I and resolved by 1% agarose gel. The gel was run for 45 min at 100 V and then photographed under ultraviolet light using the Gel Catcher Model 1500 imaging system (Taiwan Green Version Technology Ltd., New Taipei City, Taiwan).

### Cell culture

Human cervical cancer cell line HeLa-S3 (ATCC CCL-2.2) was purchased from the Bioresource Collection and Research Center, Food Industry Research and Development Institute, Hsinchu, Taiwan. HeLa-S3 cells were cultured at 37°C with 5% CO_2_ in Gibco Ham's F-12K medium (Life Technologies, Carlsbad, CA, USA) supplemented with 10% Gibco Qualified Fetal Bovine Serum (Life Technologies), 100 U/ml penicillin and 100 μg/mL streptomycin. The medium was refreshed every 3 to 4 days.

### Cell viability assay

Cell viability was determined by observation under phase contrast microscopy as well as by the ability of viable cells to reduce the yellow 3-(4,5-dimethylthiazol-2-yl)-2,5-diphenyltetrazolium bromide (MTT; Sigma-Aldrich) to purple formazan in the mitochondria. HeLa-S3 cells were seeded at 5 × 10^4^ cells/well in 24-well plates. After 48 h, cells were treated with 0 to 100 μg/ml of PEI-NH-CNTs in F-12K medium for another 48 h. Cells were fixed with 4% (*w*/*v*) paraformaldehyde for microscope observation. For MTT assay, cells were incubated in freshly prepared 1 mg/ml of MTT in PBS for 2 h. After removal of the MTT solution, dimethyl sulfoxide was added to dissolve the purple MTT formazan crystals. The absorbance of the resulting solution was quantified spectrophotometrically at 570 nm, using a reference wavelength of 630 nm.

### siRNA transfection

HeLa-S3 cells were seeded at 2 × 10^5^ cells/well in six-well plates. After 24 h, PEI-NH-CNTs (0.5 to 10 μg) was complexed with siGAPDH (0.5 μg) at various PEI-NH-CNT/siGAPDH mass ratios (1:1 to 20:1) in serum-free RPMI-1640 medium on ice for 1 h and then incubated with HeLa-S3 cells for 48 h. The final siGAPDH concentration was 30 nM. To serve as positive control, 0.5 μg siGAPDH was transfected by DharmaFECT Transfection Reagent (Thermo Fisher Scientific) according to the manufacturer's instructions.

### Real-time polymerase chain reaction

Total RNA was isolated from HeLa-S3 cells by Trizol® Reagent (Life Technologies), and reverse transcription was carried out using the Applied Biosystems High Capacity cDNA Reverse Transcription Kit (Life Technologies) according to the manufacturer's instructions. The cDNA was diluted to a final concentration of approximately 1 ng/μl and reacted with gene-specific primer pairs and Applied Biosystems SYBR® Green PCR Master Mix (Life Technologies) according to the manufacturer's protocol. The primer sequences for GAPDH (NM_002046) and β-actin (NM_001101) were designed by Origene (Rockville, MD, USA). Primer specificity was confirmed by Primer-BLAST developed at NCBI, and primer PCR efficiency was validated to be close to 100%. Genes of interest were detected and amplified by Applied Biosystems 7300 Real-Time PCR System (Life Technologies) with the following conditions: 2 min at 50°C, 10 min at 95°C, and 40 cycles of amplification at 95°C for 15 s and 60°C for 1 min, followed by melting curve analysis. Amplicons were visualized with electrophoresis on a 1.4% agarose gel to ensure the presence of a single product. The mRNA level of each gene was analyzed by the Applied Biosystems Sequence Detection Software V1.2 (Life Technologies) and normalized to that of GAPDH. Relative gene expression was calculated by the comparative Ct (2^−ΔΔct^) method [31] and expressed as fold changes (*x*-fold) relative to the control.

### Statistical analysis

Statistical analysis was performed on data from at least three independent experiments. Significant difference relative to the control was tested using Student's *t* test. Levels of significance of *p* < 0.05 and 0.01 were accepted as significant and highly significant, respectively.

## Results and discussion

### Results

#### PEI-NH-CNT suspensions

**Figure 1:**
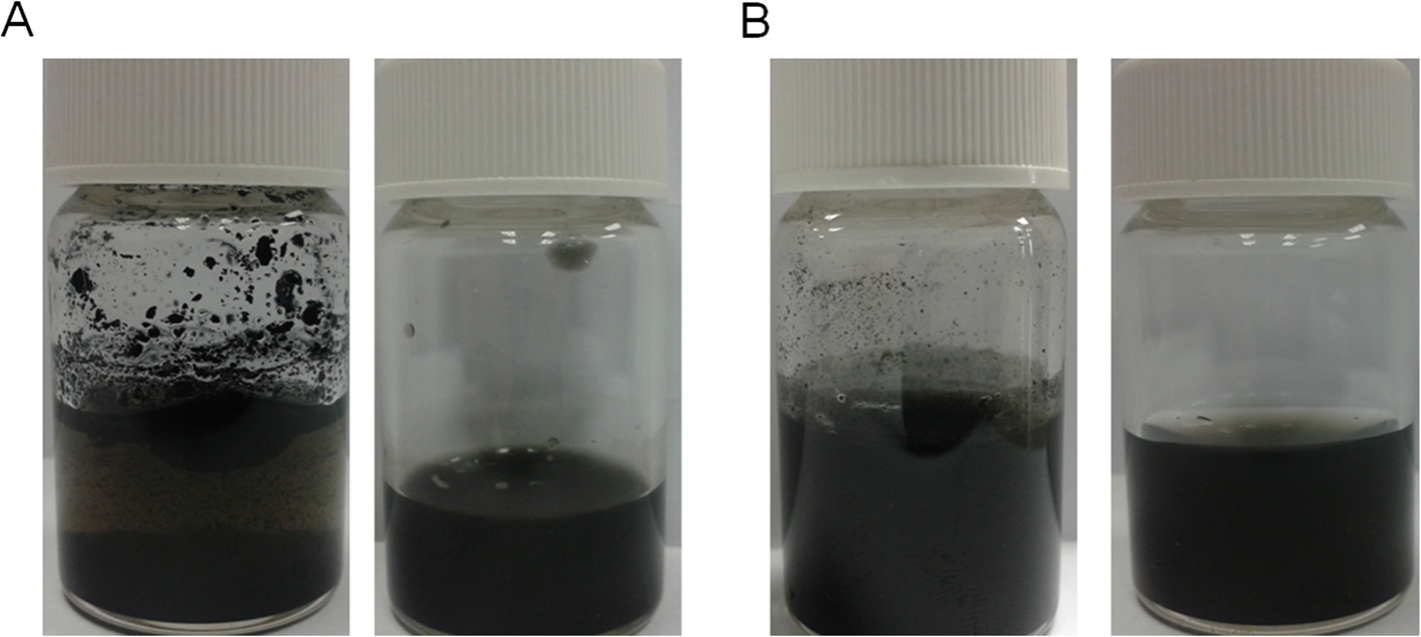
**Suspension of PEI-NH-SWNTs and PEI-NH-MWNTs in water.** PEI-NH-SWNTs (**A**) and PEI-NH-MWNTs (**B**) were solubilized in ddH_2_O at a concentration of 1 mg/ml and sonicated for 15 min (left images). Large aggregates were removed by centrifugation at 3,000 rpm for 30 min to obtain a more homogeneous suspension (right images).

PEI functionalization remarkably increased the degree of dispersibility of SWNTs and MWNTs. After being dispersed in ddH_2_O at 1 mg/ml and sonicated for 15 min, PEI-NH-MWNTs and PEI-NH-SWNTs can be solubilized in water and maintained in suspension form for over 6 months without further sonication (left images, Figure 1A, B). Because agglomeration of carbon nanotubes as a result of van der Waals' interaction tends to increase cytotoxicity [32,33], PEI-NH-CNTs were subjected to centrifugation to remove large aggregates, and the supernatant gave a more homogeneous solution of PEI-NH-CNTs for the following studies (right images, Figure 1A, B).

### Morphology of PEI-NH-CNTs

**Figure 2:**
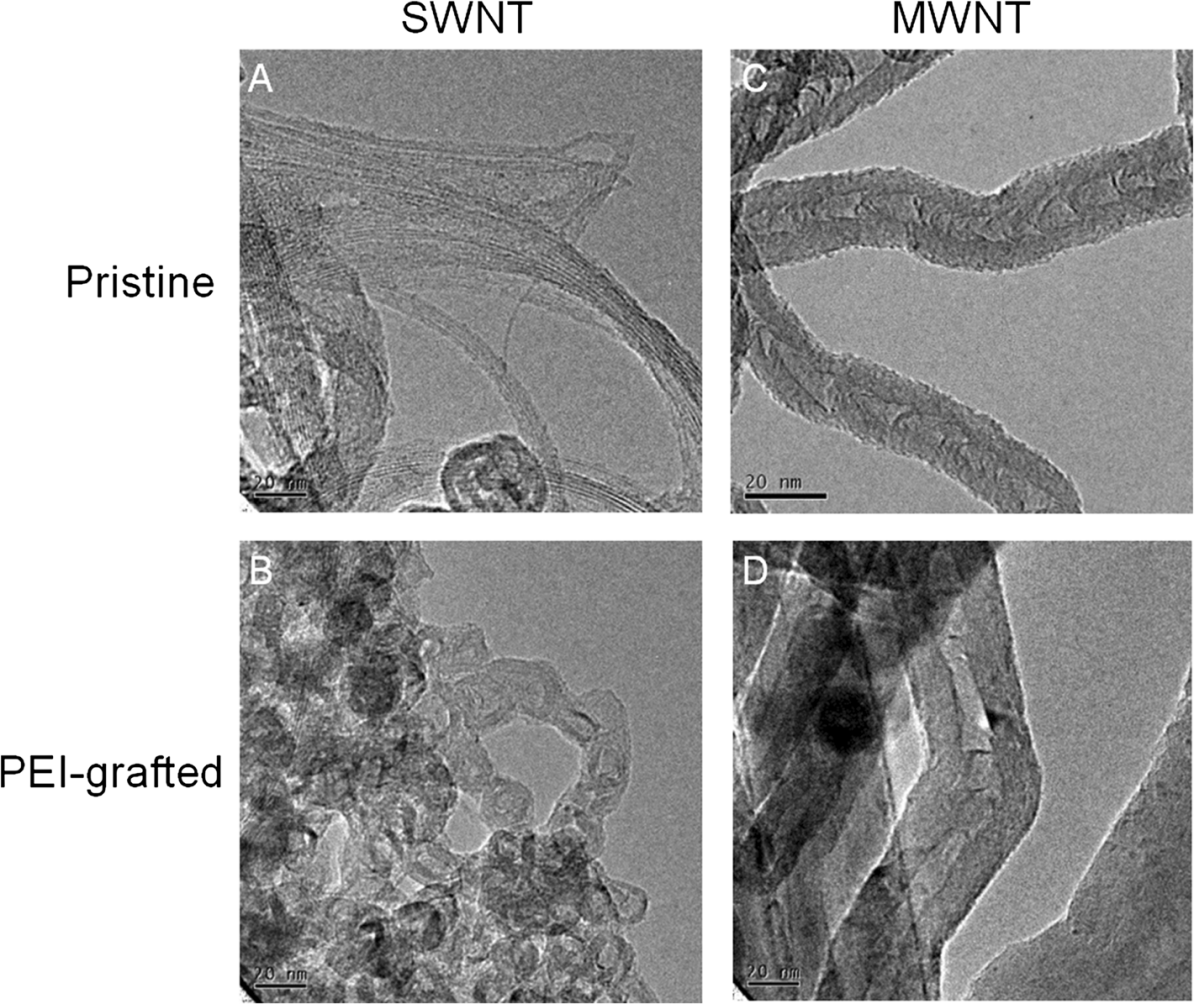
**TEM images of pristine and PEI-functionalized carbon nanotubes.** The surface morphology of pristine SWNTs (**A**) and MWNTs (**C**) was compared with that of PEI-NH-SWNTs (**B**) and PEI-NH-MWNTs (**D**) by a JEOL 2000FX TEM. Bar 20 nm.

**Figure 3:**
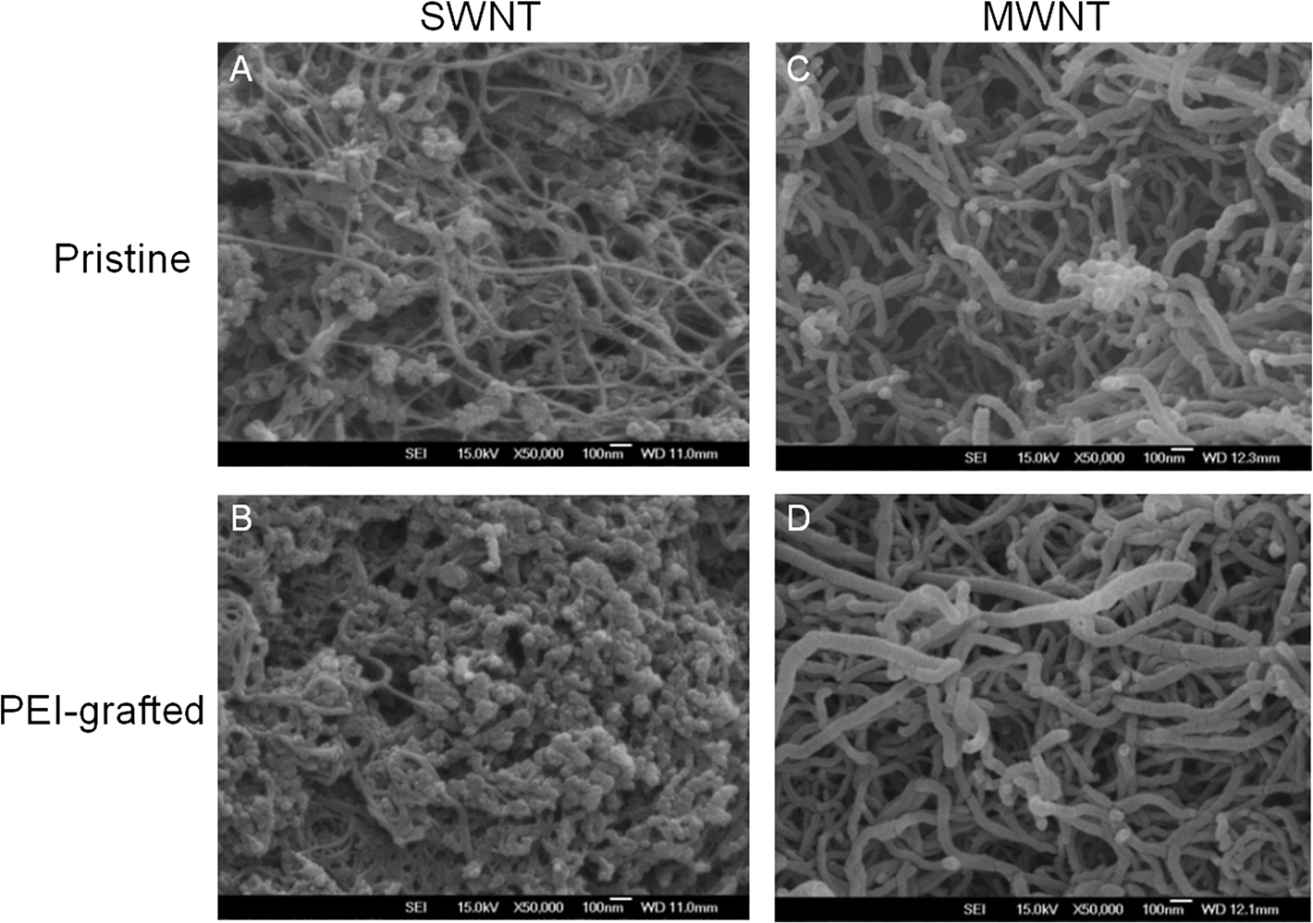
**SEM images of pristine and PEI-functionalized carbon nanotubes.** The surface morphology of pristine SWNTs (**A**) and MWNTs (**C**) was compared with that of PEI-NH-SWNTs (**B**) and PEI-NH-MWNTs (**D**) by a JSM-6500F SEM. Bar 100 nm.

The morphology of PEI-NH-CNTs compared to pristine CNTs was studied by SEM and TEM. The increase in wall thickness of PEI-NH-MWNTs compared to pristine MWNTs suggests that PEI was coated on the surface of PEI-NH-MWNTs (Figures 2C, D and 3C, D). On the other hand, the aggregates originally present in pristine SWNTs were considered as amorphous carbon (Figure 3A), but the dramatic increase in agglomerate structures on the surface of PEI-NH-SWNTs resulted from PEI modification (Figures 2A, B and 3A, B).

#### FTIR spectroscopy of PEI-NH-CNTs

**Figure 4:**
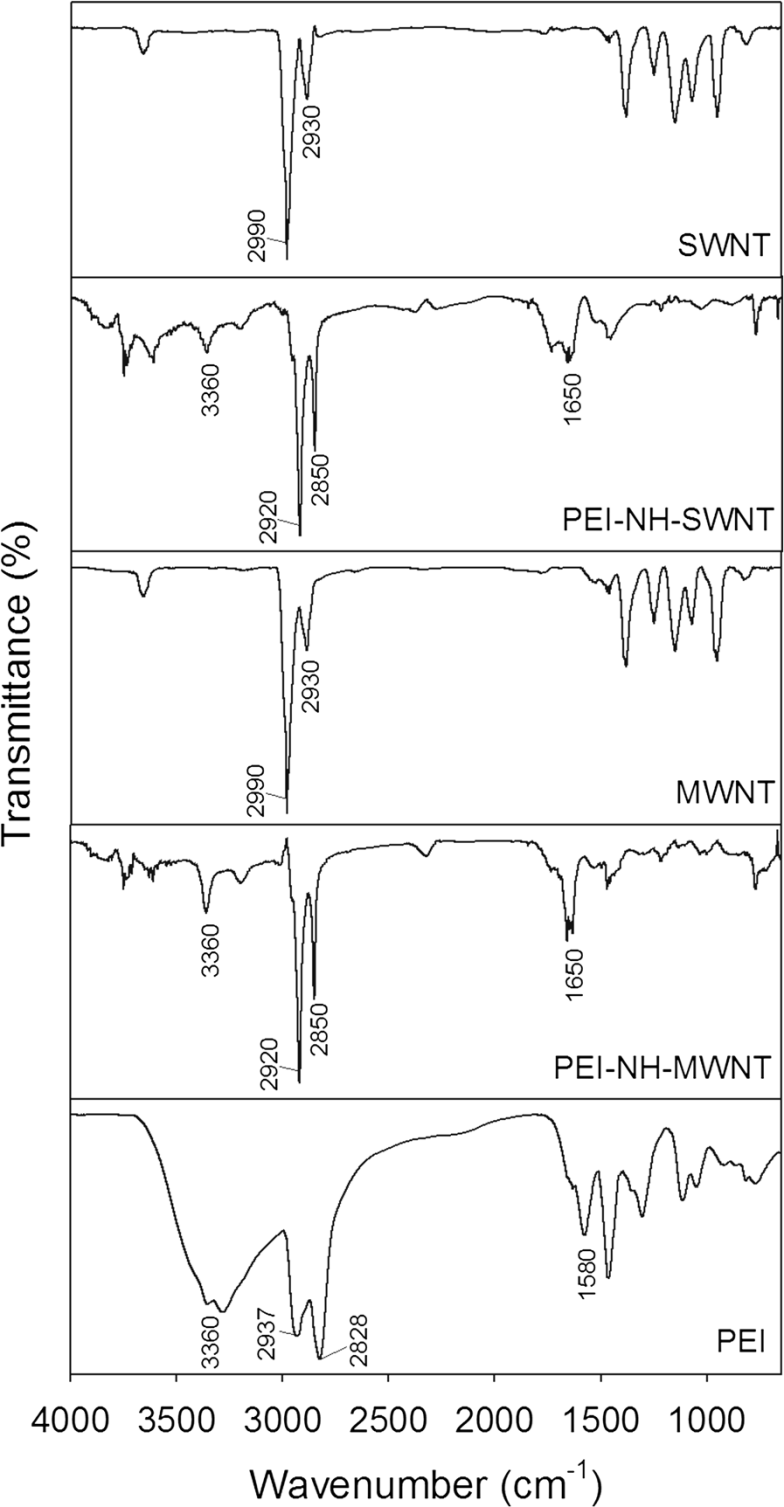
**FTIR spectra of pristine and PEI-functionalized carbon nanotubes.** Pristine and PEI-functionalized carbon nanotubes were analyzed by a PerkinElmer Spectrum 100 FTIR spectrometer, and the spectra were compared with that of pure PEI.

Binding of PEI to SWNTs or MWNTs was analyzed by FTIR spectroscopy. The characteristic peak at 3,360 cm^−1^ was assigned to N-H of PEI, which was present in PEI-NH-SWNTs and PEI-NH-MWNTs, but not in pristine SWNTs or MWNTs (Figure 4). The two major peaks at 2,990 and 2,930 cm^−1^ in pristine SWNTs and MWNTs were contributed by *sp*^2^ and *sp*^3^ carbon atoms, respectively [34], and were shifted to 2,920 and 2,850 cm^−1^ in PEI-NH-SWNTs and PEI-NH-MWNTs. Finally, the band at 1,650 cm^−1^ in the spectra of PEI-NH-SWNTs and PEI-NH-MWNTs resulted from the bending of primary amine groups (-NH_2_), which was incorporated into a broad band at 1,580 cm^−1^ in PEI.

#### PEI content of PEI-NH-CNTs

**Figure 5:**
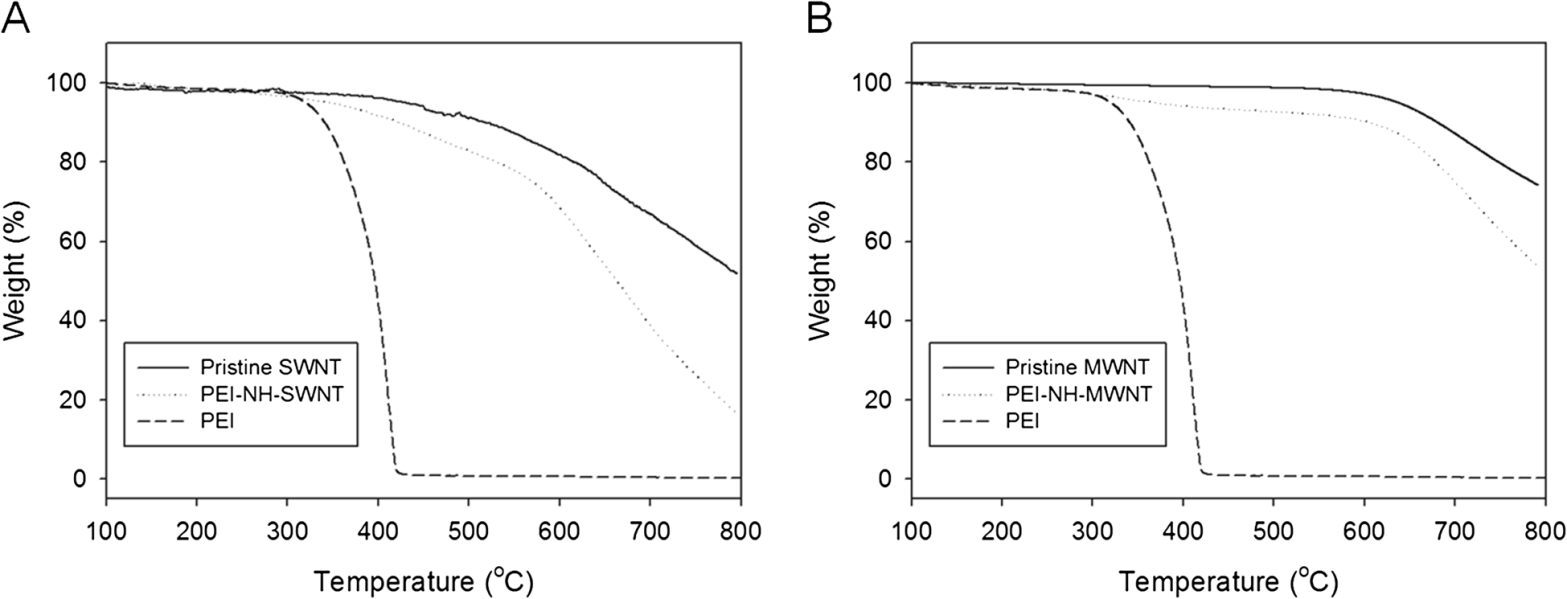
**TGA of pristine and PEI-functionalized carbon nanotubes.** The amount of PEI introduced to PEI-NH-SWNTs (**A**) or PEI-NH-MWNTs (**B**) during the functionalization procedure was quantified by the additional weight loss of PEI-NH-SWNTs and PEI-NH-MWNTs at 420°C compared to pristine carbon nanotubes.

The amount of PEI introduced to PEI-NH-CNTs during the functionalization procedure was quantified by TGA. Pure PEI degraded nearly completely at around 420°C (Figure 5). Pristine MWNTs were thermally stable up to approximately 600°C while SWNTs were relatively unstable, and weight loss was observed at temperatures over 450°C (Figure 5). The additional weight loss of PEI-NH-SWNTs and PEI-NH-MWNTs at 420°C compared to pristine carbon nanotubes was correlated directly to the mass of PEI conjugated on PEI-NH-CNTs. Consequently, the mass attributed to PEI functionalization in PEI-NH-SWNTs and PEI-NH-MWNTs was 5.08% (*w*/*w*) and 5.28% (*w*/*w*), respectively.

#### Particle size of PEI-NH-CNTs

**Figure 6:**
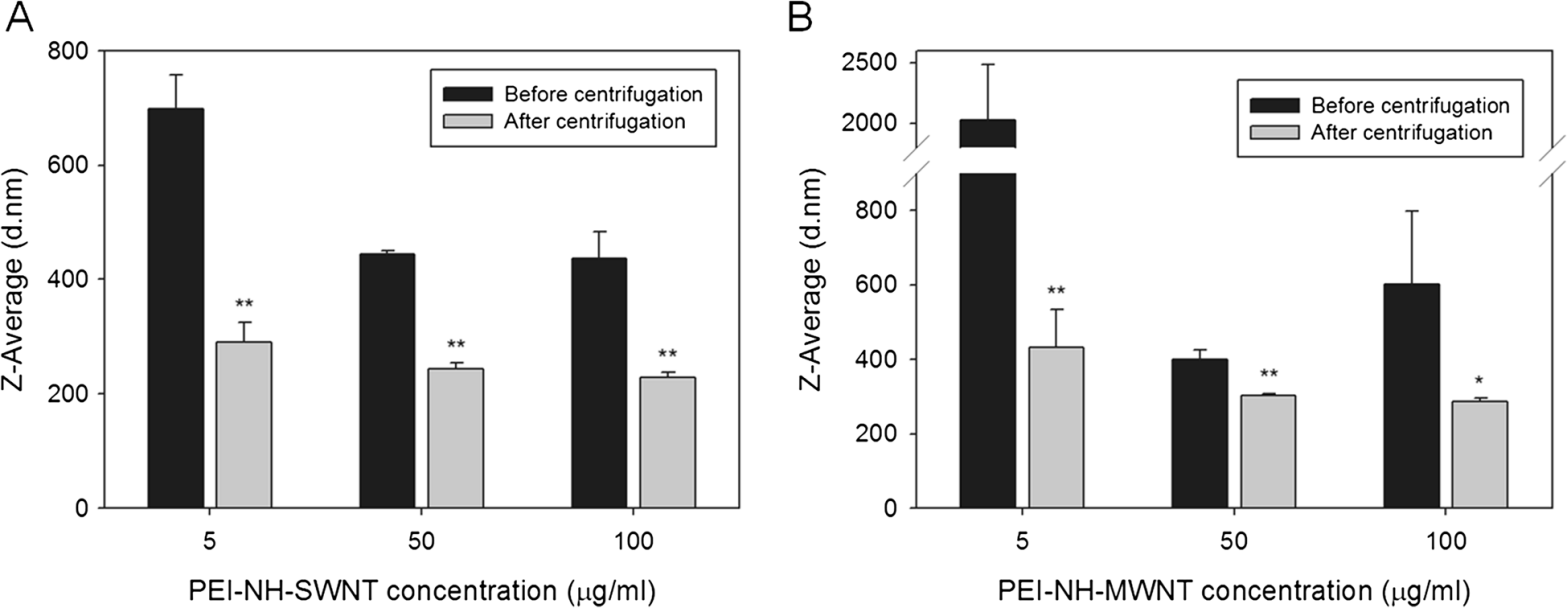
**Average particle size of PEI-NH-SWNTs and PEI-NH-MWNTs before and after centrifugation.** The average particle diameters of 5, 50, and 100 μg/ml of PEI-NH-SWNTs (**A**) or PEI-NH-MWNTs (**B**) before and after removal of large aggregates through centrifugation was analyzed by dynamic light scattering. Before centrifugation, PEI-NH-SWNTs or PEI-NH-MWNTs were solubilized in ddH_2_O at a concentration of 1 mg/ml and sonicated for 15 min; after centrifugation, PEI-NH-SWNTs or PEI-NH-MWNTs were centrifuged at 3,000 rpm for 30 min to remove large aggregates. Error bars represent standard deviations (*n* ≥ 3). *p < 0.05 and **p < 0.01 compared to PEI-NH-SWNTs or PEI-NH-MWNTs of the same concentration before centrifugation.

In order to deliver siRNAs into mammalian cells, PEI-NH-CNTs must penetrate the cell membrane. The particle size of PEI-NH-CNTs may therefore be an important factor in determining transfection efficiency. The particle size of 5 to 100 μg/ml PEI-NH-SWNTs and PEI-NH-MWNTs before and after removal of large aggregates through centrifugation was analyzed by dynamic light scattering (Figure 6). Although dynamic light scattering is usually applied to determine the diameter distribution of spherical particles, it also facilitates the understanding of size distribution of dispersed carbon nanotubes [35-38]. Prior to centrifugation, the average particle size of 5 μg/ml PEI-NH-SWNTs and PEI-NH-MWNTs was the highest among the concentrations tested, due possibly to the inhomogeneous nature of the suspension. After centrifugation, the average particle size of 5 to 100 μg/ml PEI-NH-SWNTs and PEI-NH-MWNTs in the supernatant was 229 ± 8 to 291 ± 34 and 287 ± 8 to 433 ± 102 nm, which were significantly lower than those before centrifugation (Figure 6). In addition, when the particle size of different concentrations of PEI-NH-SWNTs or PEI-NH-MWNTs was compared, no significant difference was observed. These results indicate that the centrifugation procedure effectively reduced the particle size and increased the homogeneity of PEI-NH-CNTs.

#### Zeta potential of PEI-NH-CNTs

**Figure 7:**
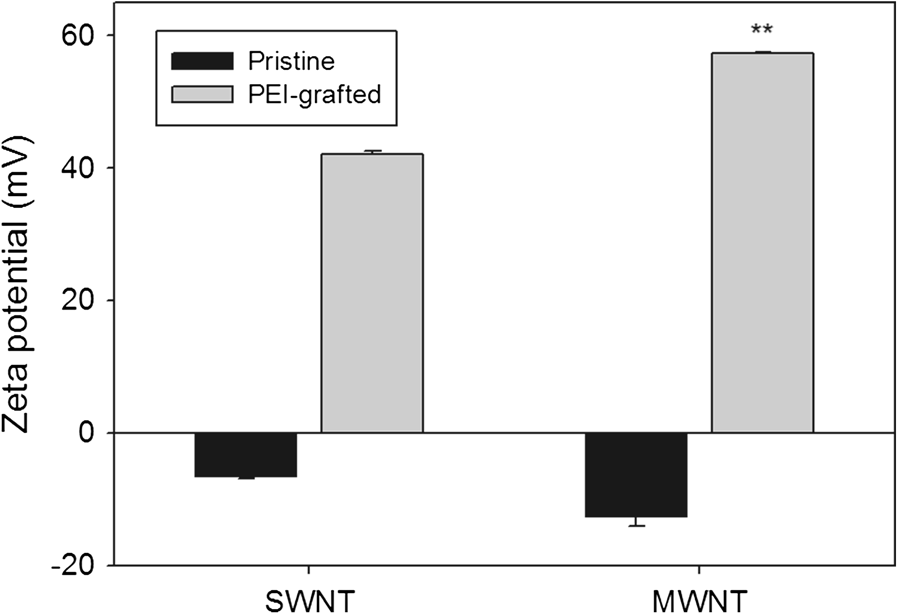
**Zeta potential of pristine and PEI-functionalized carbon nanotubes.** The zeta potential of 1 mg/ml pristine or PEI-grafted carbon nanotubes at 25°C and neutral pH was determined by dynamic light scattering. Error bars represent standard deviations (*n* ≥ 3). **p < 0.01 compared to PEI-NH-SWNTs.

The zeta potential of 1 mg/ml pristine or PEI-grafted carbon nanotubes at 25°C and neutral pH was determined through dynamic light scattering. The zeta potential of pristine SWNTs and MWNTs was negative (Figure 7), similar to those reported in the literature [39,40]. As expected, PEI functionalization increases the positive charge on the surface of PEI-NH-CNTs, resulting in positive zeta potentials, which were higher in PEI-NH-MWNTs compared to PEI-NH-SWNTs (Figure 7). The stability of PEI-NH-CNT suspension may therefore be maintained by electrostatic repulsion contributed by the cationic PEI.

#### Binding of siRNAs to PEI-NH-CNTs

**Figure 8:**
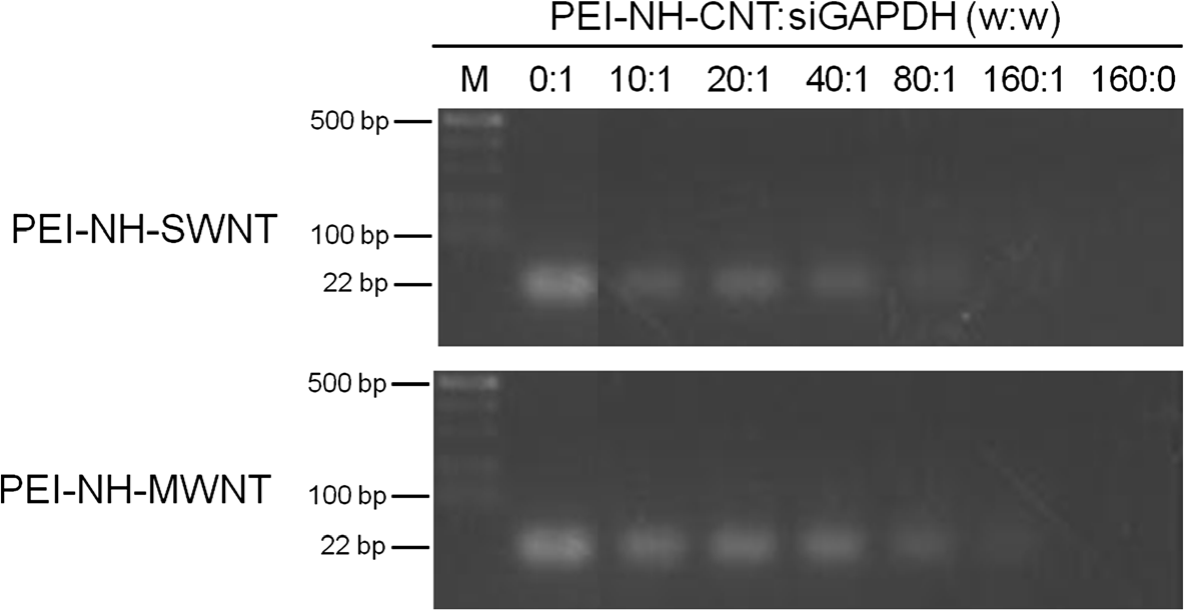
**Binding capacity of PEI-NH-SWNTs and PEI-NH-MWNTs towards siRNAs.** PEI-NH-SWNTs (upper panel) and PEI-NH-MWNTs (lower panel) were complexed with a commercially available positive control siRNA against the housekeeping gene glyceraldehyde 3-phosphate dehydrogenase (siGAPDH) at various mass ratios, followed by EMSA.

The siRNA binding capacity of PEI-NH-CNTs was studied by complexing PEI-NH-CNTs to a commercially available positive control siRNAs against the housekeeping gene GAPDH, followed by electrophoretic mobility shift assay (EMSA). The fluorescence dye SYBR Green I intercalates with free siRNAs, resulting in a 22-bp fluorescent band under gel electrophoresis. Binding of PEI-NH-CNTs to siRNAs resulted in reduced availability of siRNAs for SYBR Green I intercalation, thus reducing the fluorescence signal [18,20,21,28]. As shown in Figure 8, there was a gradual decrease in fluorescence intensity with increasing PEI-NH-CNT/siGAPDH mass ratios. The migration of siGAPDH was completely inhibited when the mass ratios of PEI-NH-SWNTs to siGAPDH and PEI-NH-MWNTs to siGAPDH were 80:1 and 160:1, respectively (Figure 8). These results indicate that both PEI-NH-SWNTs and PEI-NH-MWNTs could bind and form a stable complex with siRNAs.

#### Cytotoxicity of PEI-NH-CNTs

**Figure 9:**
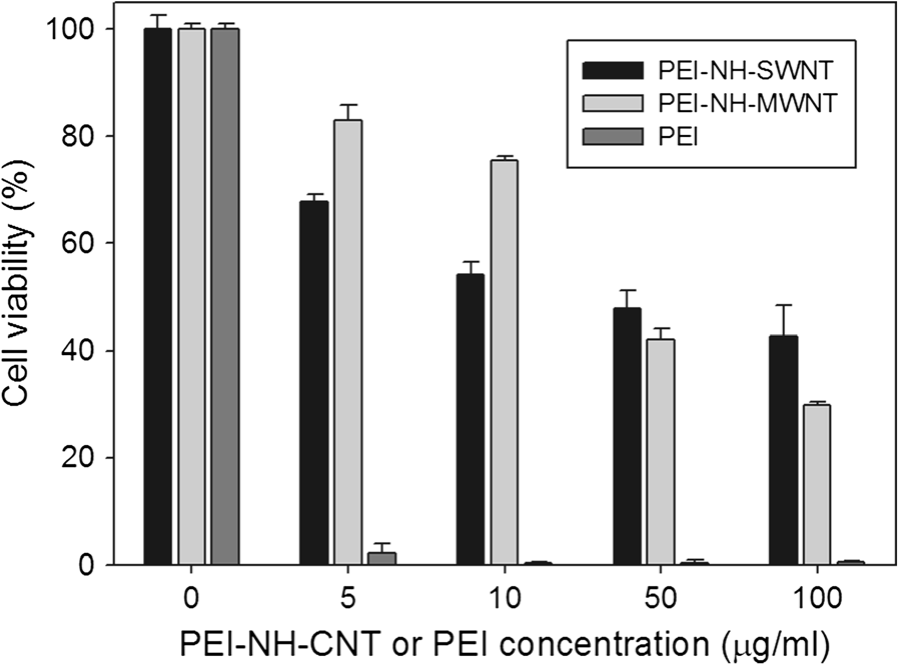
**Cytotoxicity of PEI-NH-SWNTs and PEI-NH-MWNTs compared to PEI.** Human cervical cancer cells HeLa-S3 were treated with 0 to 100 μg/ml of PEI-NH-SWNTs, PEI-NH-MWNTs, or pure PEI for 48 h. Cell viability was determined by MTT assay and expressed as the percentage of the optical density at 570 nm of treated cells relative to control cells. Error bars represent standard deviations (*n* ≥ 3). Statistical significance was observed at all concentrations of PEI-NH-SWNTs, PEI-NH-MWNTs, or pure PEI compared to the control (0 μg/ml).

Human cervical cancer cells HeLa-S3 were treated with various concentrations of PEI-NH-SWNTs or PEI-NH-MWNTs for 48 h to examine their cytotoxicity. Viability of HeLa-S3 cells decreased with increasing concentrations of PEI-NH-CNTs (Figure 9). The half-maximal inhibitory concentrations (IC_50_) of PEI-NH-SWNTs and PEI-NH-MWNTs were 23.6 and 40.5 μg/ml, respectively. On the other hand, pure PEI was relatively toxic, with an IC_50_ of 0.56 μg/ml. At a concentration of 5 μg/ml, less than 2% of cells were viable in the presence of PEI, while 70% to 80% of cells were viable when incubated with PEI-NH-SWNTs or PEI-NH-MWNTs (Figure 9). These results suggest that PEI-NH-CNTs were less cytotoxic to HeLa-S3 cells compared to PEI.

#### Transfection of siRNAs by PEI-NH-CNTs

**Figure 10:**
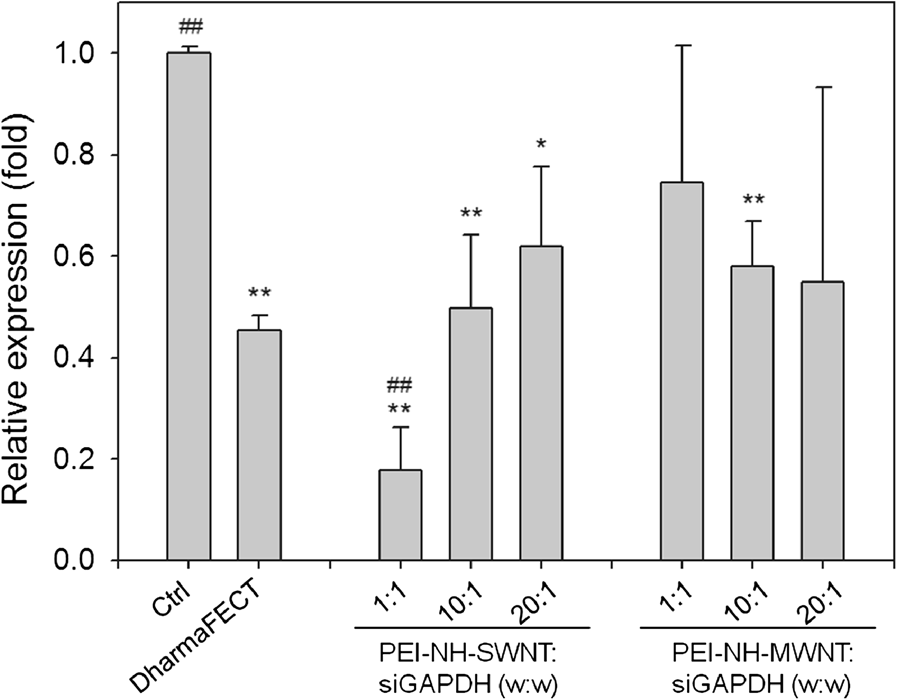
**Relative GAPDH mRNA expression of HeLa-S3 cells transfected with PEI-NH-CNT/siGAPDH complexes.** PEI-NH-SWNTs or PEI-NH-MWNTs were complexed with siGAPDH at mass ratios of 1:1, 10:1, and 20:1 and incubated with HeLa-S3 cells to achieve a final siGAPDH concentration of 30 nM. After 48 h, the mRNA level of GAPDH was analyzed by quantitative PCR. The level of GAPDH gene suppression was quantitated to evaluate the transfection efficiency of PEI-NH-SWNTs and PEI-NH-MWNTs. Control, HeLa-S3 cells cultured in growth medium for 48 h; DharmaFECT, HeLa-S3 cells transfected with siGAPDH using DharmaFECT as transfection reagent. Error bars represent standard deviations (*n* ≥ 3). *p < 0.05 and **p < 0.01 compared to the control; ^##^*p* < 0.01 compared to DharmaFECT.

PEI-NH-CNTs were complexed with siGAPDH at mass ratios of 1:1, 10:1, and 20:1 and incubated with HeLa-S3 cells to achieve a final siGAPDH concentration of 30 nM. After 48 h, transfection efficiency of PEI-NH-CNTs was evaluated by the mRNA level of GAPDH and was compared with that of DharmaFECT. Transfection of siGAPDH with DharmaFECT resulted in more than 50% suppression of the mRNA level of GAPDH (Figure 10). Delivery of siGAPDH by PEI-NH-SWNTs suppressed GAPDH mRNA expression to 18%, 50%, and 62% of untreated control at PEI-NH-SWNT/siGAPDH ratios of 1:1, 10:1, and 20:1, respectively. On the other hand, GAPDH gene expression was reduced to 55% to 75% of control when siRNAs were delivered by PEI-NH-MWNTs. However, statistical significance (*p* < 0.01) was only observed at PEI-NH-MWNT/siGAPDH ratio of 10:1 (Figure 10). Compared to DharmaFECT, PEI-NH-SWNTs gave rise to more significant suppression of GAPDH gene expression at a PEI-NH-SWNT/siGAPDH mass ratio of 1:1. There was no significant difference between the transfection efficiency of PEI-NH-SWNTs and PEI-NH-MWNTs except when the PEI-NH-CNT/siGAPDH ratio was 1:1 (Figure 10). These results suggest that PEI-NH-SWNTs and PEI-NH-MWNTs successfully delivered siGAPDH to HeLa-S3 cells and that the siRNA transfection efficiency of PEI-NH-SWNTs and PEI-NH-MWNTs was comparable to that of DharmaFECT.

### Discussion

Previous studies have utilized a similar direct amination procedure as in this report to produce PEI-grafted MWNTs. Varkouhi et al. modified MWNTs of 9.5 nm in diameter with 25-kDa branched PEI, while Foillard et al. synthesized PEI-functionalized MWNTs with the less cytotoxic 600-Da branched PEI [21,28]. In both studies, MWNTs were shortened by ultrasonication prior to PEI functionalization. This study applied direct amination method to both SWNTs and MWNTs but without shortening the carbon nanotubes. PEI functionalization increased the solubility of SWNTs and MWNTs in water as well as their binding affinity for siRNAs. We removed larger aggregates of PEI-NH-SWNTs and PEI-NH-MWNTs by centrifugation [21,28,41] to improve their dispersity and homogeneity (Figure 1). After centrifugation, the particle size of PEI-NH-SWNTs and PEI-NH-MWNTs was decreased and was less affected by concentration (Figure 6). Surface modification of carbon nanotubes by PEI can be observed through TEM, SEM, and FTIR spectroscopy (Figures 2, 3, and 4) as well as the dramatic change in zeta potentials (Figure 7), and the amount of grafted PEI was estimated by TGA (Figure 5). Although both PEI-NH-SWNTs and PEI-NH-MWNTs caused HeLa-S3 cell deaths in a dose-dependent manner, they were less cytotoxic compared to pure PEI (Figure 9). PEI-NH-SWNTs and PEI-NH-MWNTs were capable of binding siRNAs (Figure 8) and delivering them into HeLa-S3 cells, resulting in suppression of the mRNA level of GAPDH to an extent similar to that of DharmaFECT, the commercial transfection reagent for siRNAs (Figure 10).

It was reported that the cytotoxicity of PEI-grafted MWNTs is higher than 25-kDa PEI alone in human lung cancer cells (H1299), suggesting that MWNTs enhance the cytotoxicity of PEI [28]. Studies on *Daphnia magna* also demonstrated that PEI coating increased MWNT toxicity, which was associated with the size of PEI coating, but not the surface charge of PEI [42]. In contrast, our results suggest that cell viability was higher in the presence of PEI-NH-SWNTs and PEI-NH-MWNTs compared to pure 25-kDa PEI (Figure 9). Liu et al. applied a different approach to obtain PEI-grafted MWNTs but reached a similar conclusion to this study by demonstrating that, at concentrations higher than 15 μg/ml, 25-kDa PEI alone is more toxic to 293, HepG2, and COS7 cells compared to PEI-grafted MWNTs [23]. In addition, Wang et al. indicated that PEI-functionalized SWNTs exhibited no significant cytotoxicity to PC-3 cells at concentrations lower than 30 μg/ml but may lead to an increase in apoptosis [24]. In addition to concentration, cytotoxicity of carbon nanotubes is correlated with the type of functionalization [43,44], the degree of agglomeration [32,33], as well as nanotube length [45]. Pathways leading to carbon nanotube cytotoxicity were mainly related to DNA damage and the induction of reactive oxygen species [46]. Nevertheless, due to the difference in the types and synthetic procedures of PEI-functionalized carbon nanotubes between this and previous studies and the tolerance of various cells or tissues to the nanomaterial, the cause of carbon nanotube cytotoxicity remains to be investigated.

Results from EMSA showed that at PEI-NH-SWNT/siGAPDH and PEI-NH-MWNT/siGAPDH mass ratios of 80:1 and 160:1, respectively, siGAPDH was completely complexed with PEI-NH-CNTs (Figure 8). However, suppression of GAPDH mRNA expression was observed at relatively lower mass ratios of 1:1 to 1:20 (Figure 10). Such discrepancy in the effective ratios of functionalized carbon nanotubes to siRNAs or DNAs in EMSA and in gene delivery is also presented in previous studies [18,20,23]. Amino-functionalized MWNTs (MWNT-NH_3_^+^) is unable to completely retard the migration of siRNAs in EMSA at a MWNT-NH_3_^+^/siRNA mass ratio of 80:1, but the cationic MWNTs successfully delayed tumor growth in animal models when complexed with siRNAs at a mass ratio of 8:1 [20]. These findings implicate that complete binding of siRNAs by PEI-NH-CNTs may not be necessary for a successful intracellular siRNA delivery. Increasing the amount of PEI-NH-CNTs relative to siRNAs may provide more stable complexes of PEI-NH-CNT/siRNA but may possibly hinder the dissociation of siRNAs from PEI-NH-CNTs once the complex enters the cytosol.

Carbon nanotubes are considered an efficient carrier for nonviral gene delivery. Compared to viral vectors, nonviral transfection reagents eliminate the possibility of carcinogenesis and severe immune response caused by viral vectors and provide a safer alternative for the clinical application of gene therapy [47]. However, the efficiency of nonviral transfection is relatively low compared to viral transfection. We showed that the siRNA transfection efficiency of both PEI-NH-SWNTs and PEI-NH-MWNTs was comparable to the commercially available DharmaFECT reagent (Figure 10). A similar comparison of transfection efficiency with another common transfection reagent was reported on MWNTs functionalized with 600-Da PEI [21]. Nevertheless, Varkouhi et al. compared the transfection efficiency of PEI-functionalized MWNTs with Lipofectamine but found that PEI-functionalized MWNTs were less effective in siRNA delivery [28]. Further studies on *in vivo* siRNA transfection by PEI-functionalized carbon nanotubes may be necessary to elucidate their effectiveness in gene delivery.

## Conclusions

This study demonstrated that effective carrier for siRNAs can be achieved through direct amination of SWNTs and MWNTs with 25-kDa branched PEI. The resulting PEI-NH-SWNTs and PEI-NH-MWNTs complexed with siRNAs, successfully delivered siRNAs into HeLa-S3 cells, and exhibited transfection efficiency comparable to commercial reagents. Modification of the PEI functionalization procedure may be required to reduce the cytotoxicity of PEI-NH-SWNTs and PEI-NH-MWNTs. Further investigation on the *in vivo* transfection efficiency of PEI-NH-SWNTs and PEI-NH-MWNTs is necessary to enhance their therapeutic potential in gene therapy.

## Competing interests

The authors declare that they have no competing interests.

## Authors’ contributions

YPH and IJL carried out the experiments. YPH and MJL designed the study. CCC, CCC, and YCH performed data analysis and statistical analysis. MJL drafted and finalized the manuscript. All authors read and approved the final manuscript.
